# Discoidal HDL and apoA-I-derived peptides improve glucose uptake in skeletal muscle

**DOI:** 10.1194/jlr.M032904

**Published:** 2013-05

**Authors:** Jonathan Dalla-Riva, Karin G. Stenkula, Jitka Petrlova, Jens O. Lagerstedt

**Affiliations:** Department of Experimental Medical Science, Lund University, S-221 84 Lund, Sweden

**Keywords:** muscle fiber, GLUT4 transporter, diabetes, insulin resistance, apolipoprotein A-I, high density lipoprotein

## Abstract

Lipid-free apoA-I and mature spherical HDL have been shown to induce glucose uptake in skeletal muscle. To exploit apoA-I and HDL states for diabetes therapy, further understanding of interaction between muscle and apoA-I is required. This study has examined whether nascent discoidal HDL, in which apoA-I attains a different conformation from mature HDL and lipid-free states, could induce muscle glucose uptake and whether a specific domain of apoA-I can mediate this effect. Using L6 myotubes stimulated with synthetic reconstituted discoidal HDL (rHDL), we show a glucose uptake effect comparable to insulin. Increased plasma membrane GLUT4 levels in ex vivo rHDL-stimulated myofibers from HA-GLUT4-GFP transgenic mice support this observation. rHDL increased phosphorylation of AMP kinase (AMPK) and acetyl-coA carboxylase (ACC) but not Akt. A survey of domain-specific peptides of apoA-I showed that the lipid-free C-terminal 190–243 fragment increases plasma membrane GLUT4, promotes glucose uptake, and activates AMPK signaling but not Akt. This may be explained by changes in α-helical content of 190–243 fragment versus full-length lipid-free apoA-I as assessed by circular dichroism spectroscopy. Discoidal HDL and the **190–243** peptide of apoA-I are potent agonists of glucose uptake in skeletal muscle, and the C-terminal α-helical content of apoA-I may be an important determinant of this effect.

Apolipoprotein A-I (apoA-I) is the primary protein component of high-density lipoprotein (HDL) and as such is important for reverse cholesterol transport (1). The apoA-I protein exists in a variety of structural organizations in the different forms of HDL and in the lipid-free state (2). The lipid-bound forms include both discoidal planar HDL particles of different diameters and mature spherical HDL particles of varying sizes and lipid compositions (**Fig. 1**). Key features of apoA-I that determine its function include high structural plasticity resulting in major changes in secondary, tertiary, and quaternary structures between apo- and lipid-bound states, along with an amphipathic character of the helices formed by lipid association. It is known that reduced plasma HDL is an independent risk factor for cardiovascular disease (3) and type 2 diabetes (4), with diabetic patients having an increased risk for cardiovascular complications (5). Such outcomes are typically regarded as secondary to diabetes; however, recent data show that apoA-I in HDL directly contributes to peripheral glucose metabolism (6–9). How the various conformations of apoA-I contribute to this effect is yet to be fully clarified.

**Fig. 1.**
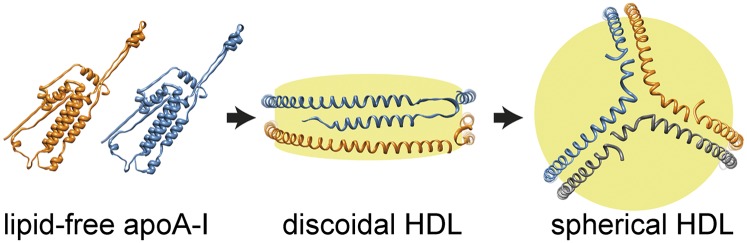
Structural models of apoA-I in lipid-free (left) (36), discoidal HDL (middle) (16), and spherical HDL (right) (37) states, indicating significant structural rearrangements in HDL maturation. The individual apoA-I molecules are shown in orange, blue, and gray, respectively. Yellow indicates the core of phospholipids, cholesterol, and cholesteryl esters.

The rate of glucose uptake in skeletal muscle, the principal site for plasma glucose clearance, is determined by cell surface levels of GLUT4, which is controlled by both the insulin signaling pathway and the AMP kinase (AMPK) contraction-induced pathway (10). As these are largely independent signaling routes, AMPK represents a therapeutic target for the maintenance of plasma glucose despite insulin resistance (11). This is exemplified by impaired contraction-induced glucose uptake in skeletal muscle of mice expressing dominant negative AMPK (12) and improved glucose control in diabetic subjects during an acute bout of exercise (13). It has been shown that lipid-free apoA-I and spherical HDL can induce glucose uptake in C2C12 myotubes (6) and in human myotubes differentiated from muscle satellite cells from diabetic donors (7) via the activation of AMPK, which provides promise for apoA-I/HDL as a novel diabetic treatment.

Despite these findings, it is not clear whether discoidal HDL is also capable of specifically regulating muscle glucose uptake and whether this occurs via AMPK. Given that HDL subspecies interact differently with cellular receptors at the vascular wall for cholesterol efflux and that discoidal HDL is a potent structure for this interaction (14), we hypothesized that discoidal HDL would be highly effective in the stimulation of glucose uptake in muscle.

Herein, we investigated the effects of synthetic discoidal HDL (rHDL) and apoA-I-derived peptides on glucose uptake, intracellular signaling, and GLUT4 translocation to the plasma membrane using L6 myotubes and flexor digitorum brevis (FDB) fibers. We show that rHDL produces insulin-like effects in these models, and we identify a novel peptide candidate that induces responses comparable to those of rHDL.

## METHODS

### Cell culture

L6 myoblasts (ATCC #CRL-1458) were grown in α-MEM (Invitrogen) supplemented with 10% FBS (Sigma) and 1% antibiotic/antimycotic (penicillin, streptomycin, amphotericin B; Invitrogen). Differentiation to myotubes was achieved by switching from growth media to 2% FBS α-MEM for 6–12 days. Cells were maintained at 37°C and 5% CO_2_.

### Expression and purification of recombinant human apoA-I and apoA-I variants

Human apoA-I variants (full-length and truncated variants produced by site-directed mutagenesis, corresponding to amino acids 1–243 and to amino acids 1–189, 44–189, 44–243, and 190–243, respectively) were expressed in *Escherichia coli* strain BL21 Star (DE3)pLysS cells (Invitrogen) from the human apoA-I gene containing a hexa-His affinity tag at the N-terminus (15). Briefly, the gene (full-length or truncated variants of the gene) was cloned into the pEXP-5 plasmid (Novagen Inc.), transferred into the bacteria, and cultivated at 37°C in LB medium with 50 µg/ml of ampicillin and 34 µg/ml of chloramphenicol. Protein expression was induced for 3–4 h following the addition of 0.5 mmol/l isopropyl-β-thiogalactopyranoside (Sigma). Following cell disruption, apoA-I was purified from the soluble fraction of the cells using a His-Trap-Nickel-chelating column (GE Healthcare) and a mobile phase of phosphate-buffered saline (PBS), pH 7.4, with 3 mol/l guanidine. The protein was then washed in PBS (pH 7.4) containing 100 mmol/l imidazole, and then eluted with PBS containing 500 mmol/l imidazole. Imidazole was removed from the protein sample by using desalting columns (GE Healthcare) equilibrated with PBS, pH 7.4. Protein purity was analyzed by SDS-PAGE, and concentration was determined by the BCA method (Pierce) or using a nanodrop 2000c spectrophotometer (Thermo Scientific).

### Production of reconstituted HDL

1-palmitoyl-2-oleoyl-*sn*-glycero-3-phosphocholine (POPC) or 1,2-dimyristoyl-*sn*-glycero-3-phosphocholine (DMPC) (Avanti Polar Lipids) was dissolved in chloroform:methanol (3:1), which was evaporated under a stream of nitrogen gas, and the resulting lipid film was resuspended in PBS. For POPC rHDL, deoxycholate was added to the POPC emulsion at a 2:1 molar ratio (deoxycholate:POPC) and incubated with apoA-I at a 156:1 molar ratio (phospholipid:protein) for 1 h at 22°C (16). Deoxycholate was removed from the POPC rHDL preparation by extensive dialysis against PBS. DMPC rHDL was prepared according to Ref. 17. Briefly, the DMPC emulsion was passed through a polycarbonate membrane with 100 nm pore size using the LiposoFast system (Avestin) a minimum of 20 times. The resulting vesicles were incubated with apoA-I at a 156:1 molar ratio (phospholipid:protein) for 4 days at 22°C. ApoA-I dimers, indicative of rHDL formation, was confirmed by blue native PAGE (Invitrogen). POPC vesicles were prepared by first passing the POPC emulsion through a polycarbonate membrane with 400 nm pore size followed by passage through a 100 nm pore size membrane using the LiposoFast system (Avestin) a minimum of 20 times. Treatments were performed with POPC rHDL unless otherwise indicated.

### Circular dichroism spectroscopy

Circular dichroism (CD) measurements were performed on a Jasco J-810 spectropolarimeter equipped with a Jasco CDF-426S Peltier set to 25°C. ApoA-I (full-length and 190–243 fragment) was diluted to 0.1 mg/ml in PBS (final concentration was 25 mmol/l phosphate, 25 mmol/l NaCl, pH 7.4), placed in a 0.1 mm quartz cuvette, and after extensive purging with nitrogen, scanned in the region 200–260 nm (scan speed was 20 nm/min). Averages of five scans were baseline-subtracted (PBS buffer; 25 mmol/l phosphate, 25 mmol/l NaCl), and the α-helical content was calculated from the molar ellipticity at 222 nm as previously described (18).

### Western blotting

Prior to stimulation, cultured cells were serum starved for 4 h in serum-free α-MEM, and all subsequent treatments, including insulin or phenformin as positive controls (Sigma), were performed in serum-free α-MEM. After treatments, cells were washed with ice-cold PBS and lysed on ice using a nondenaturing lysis buffer (1% Triton X-100, 50 mmol/l Tris, 150 mmol/l NaCl, pH 8.0) containing protease and phosphatase inhibitors (Roche). Lysates were centrifuged at 16,000 *g*, 20 min at 4°C, and then BCA protein assay (Pierce) was performed on supernatants. Equal protein amounts were separated by SDS-PAGE and transferred to nitrocellulose membranes. pAMPK, AMPK, pACC, pAkt, Akt (Cell Signaling), and tubulin (Sigma) were used for immunodetection with IRDye 800CW and 680RD secondary antibodies (LI-COR). Blots were imaged using the Odyssey Fc system and quantified using Image studio v2.0 software.

### Glucose uptake measurements

Prior to stimulation cells were starved for 2 h in serum-free α-MEM, and all subsequent treatments, which were performed in triplicate and included cytochalasin B (Sigma) as a measure of cell-associated nonspecific radioactivity, were performed in uptake buffer (140 mmol/l NaCl, 20 mmol/l HEPES, 5 mmol/l KCl, 2.5 mmol/l MgSO_4_, 1 mmol/l CaCl_2_, pH 7.4). After stimulation, treatments were replaced with 10 µmol/l 2-deoxy-D-glucose (Sigma) and 1 µCi/ml 2-[^3^H]deoxy-D-glucose (Perkin Elmer) in uptake buffer for 15 min at room temperature. Cells were then washed twice with ice-cold PBS and lysed with 1 mol/l NaOH on ice. Lysates were collected and radioactivity was quantified by scintillation counting.

### Dissection and immunostaining of skeletal muscle

Two to five transgenic mice (C57/Bl6; 10–14 weeks old) with muscle-specific HA-GLUT4-GFP expression (gift from S. Cushman, Lund University Diabetes Centre, Sweden) (19, 20), were used for each condition. The animals were euthanized, and FDB muscles dissected out and incubated with oxygenated Krebs-Hensleit carbonate Hepes (KRBH) buffer (6 mmol/l KCl, 1 mmol/l Na_2_HPO_4_, 0.2 mmol/l NaHPO_4_, 1.4 mmol/l MgSO_4_, 1 mmol/l CaCl_2_, 128 mmol/l NaCl, 10 mmol/l HEPES, pH 7.4) with 0.5% (w/v) BSA. After dissection, muscles were continually oxygenated with 95% O_2_ / 5% CO_2_ and incubated at 37°C for 2 h in a water bath with slow shaking. After incubation, muscles were washed three times with oxygenated KRBH and were then either treated with insulin (100 nmol/l), apoA-I (full-length, lipid-free), rHDL, or apoA-I fragment 190–243 or kept basal for 1 h. After stimulation, basal (nonstimulated) and stimulated muscles were fixed for 10 min with 4% paraformaldehyde in PBS, washed three times with PBS containing 1% BSA, and incubated for 30–60 min with anti-HA (Covance), followed by 30 min with fluorescently labeled secondary antibodies ALEXA-647 (Invitrogen).

### Confocal microscopy

Fixed cells were imaged using a confocal LSM 510 microscope (Zeiss) using a 40× objective, NA 1.3, using BP 505-530 and LP 650. Images were collected with the LSM software.

### Statistical analysis

All data are displayed as mean ± SEM unless indicated otherwise. Where appropriate, analysis was performed by two-tailed Student *t*-test or one-way ANOVA with Bonferroni's post hoc test using Microsoft Excel and Graph Pad Prism software. *P* ≤ 0.05 was considered significant.

## RESULTS

### rHDL induces glucose uptake and GLUT4 translocation in skeletal muscle cells

To investigate the effect of discodial HDL (rHDL) on GLUT4 translocation and glucose uptake, we produced recombinant human apoA-I and reconstituted HDL needed for cell incubations.

**Fig. 2.**
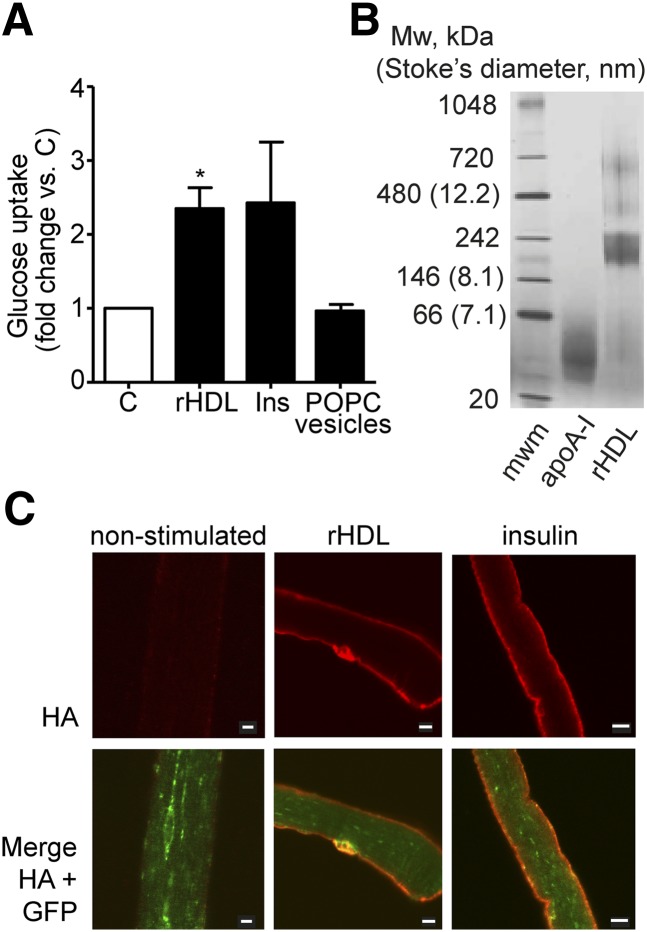
Discoidal HDL is effective at inducing glucose uptake and translocation of GLUT4 glucose transporter in muscle. (A) Glucose uptake in L6 myotubes. After 2 h serum starvation, L6 myotubes were stimulated with 2 µmol/l (60 µg/ml) POPC rHDL (expressed as total protein concentration of apoA-I in rHDL), insulin (100 nmol/l), 100 nm POPC vesicles (0.10 mmol/l), or nonstimulated (C), for 60 min before glucose uptake was determined (n = 3) *P ≤ 0.05. (B) Coomassie-stained blue native PAGE showing lipid-free apoA-I (4.5 µg) and POPC rHDL (4.9 µg apoA-I). (C) Changes in HA-GLUT4-GFP localization after 60 min treatment of FDB muscle fibers with 1 µmol/l rHDL (30 µg/ml apoA-I), 100 nmol/l insulin, or no stimulation. Myofibers were fixed and immunofluorescence performed. Size bars are 10 µm. Images are representative of three separate experiments each with myofibers from 2–4 animals.

L6 myotubes were incubated with 2 µmol/l (60 µg/ml) discoidal rHDL (expressed as total protein concentration of apo A-I; given two apoA-I molecules per particle, this corresponds to 1 µmol/l discodial rHDL) for 1 h. The rHDL treatment induced a glucose uptake that was 2.3 ± 0.39-fold (*P* ≤ 0.05) over basal, which was similar to insulin stimulation (2.4 ± 1.0-fold) (**Fig. 2A**). To test for the contribution of the constituent phospholipid to rHDL-induced glucose uptake, rHDL made with POPC was compared with 100 nm POPC vesicles containing no apoA-I protein. Incubation with empty POPC vesicles (0.10 mmol/l) did not induce glucose uptake (Fig. 2A). DMPC as the lipid constituent in protein-free lipid vesicles and as the phospholipid constituent of rHLD was also tested. Whereas rHDL particles synthesized from apoA-I (30 µg/ml) and DMPC (0.16 mmol/l) induced glucose uptake to a level similar to insulin-stimulated cells, DMPC vesicles alone did not stimulate glucose uptake (results not shown).

Blue native PAGE was performed on all rHDL preparations to confirm the formation of 10 nm diameter discoidal apoA-I dimers. Fig. 2B is a representative Coomassie-stained gel that shows monomeric lipid-free apoA-I (∼28 kDa) and the size of POPC rHDL (∼10 nm diameter). These data clearly show that rHDL exerts a potent effect on glucose uptake in muscle and that the rHDL-mediated glucose uptake is apoA-I protein dependent.

To support the glucose uptake observations, the ability of rHDL to increase the amount of GLUT4 in the plasma membrane was assessed by immunofluorescence microscopy of intact FDB muscle fibers, isolated from a transgenic mouse model with muscle-specific HA-GLUT4-GFP expression (20). The HA epitope present on the first exofacial loop of the HA-GLUT4-GFP construct allows detection of GLUT4 inserted into the plasma membrane. Intact FDB fibers were incubated ex vivo with rHDL, followed by fixation and HA antibody labeling of nonpermeabilized cells. Both insulin and rHDL treatment induced translocation of GLUT4 into the sarcolemma plasma membrane as detected by HA signal (Fig. 2C, upper panel). The lower panel in Fig. 2C displays total GLUT4 detected by GFP signal merged with the HA signal in nonstimulated and stimulated muscle fibers. Due to steric hindrance, labeling of the transverse tubules was limited, and therefore, the plasma membrane GLUT4 translocation was assessed only at the sarcolemma.

### Phosphorylation of AMPK and ACC, but not Akt, is increased in L6 myotubes treated with rHDL

**Fig. 3.**
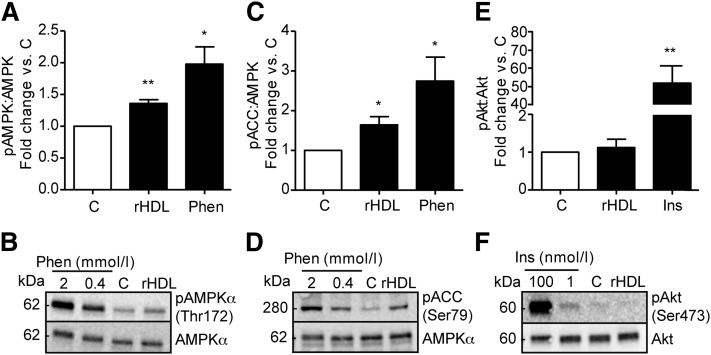
Effect of rHDL on phosphorylation of AMPK, ACC and Akt in L6 myotubes. Myotubes were serum starved for 4 h then treated for 60 min with rHDL (14 µmol/l apoA-I in rHDL), 2 mmol/l or 0.4 mmol/l phenformin, 100 nmol/l or 1 nmol/l insulin, or control (C). Densitometric analysis of lysates subjected to western blot analysis using antibodies for (A) phospho-AMPK and AMPK, (C) phospho-ACC and AMPK, and (E) phospho-Akt and Akt. (B, D, F) Representative western blots. n = 3, ± SEM; *P ≤ 0.05, **P ≤ 0.01.

The effect of apoA-I on muscle has previously been suggested to occur through a noninsulin-dependent signal pathway as described in studies using lipid-free apoA-I (6) and apoA-I in mature plasma HDL (7). To dissect the effect of discoidal rHDL on signaling pathways, we conducted western blotting using lysates from L6 myotubes incubated with rHDL. After 60 min of treatment (14 µmol/l apoA-I in rHDL), L6 myotube lysates showed increased (1.36 ± 0.071-fold; *P* ≤ 0.01) levels of phosphorylated AMPK (**Fig. 3A, B**) and its downstream target ACC (1.64 ± 0.26-fold; *P* ≤ 0.05) (Fig. 3C, D). Phenformin was used as a positive control, inducing a 1.98 ± 0.33-fold (*P* ≤ 0.05) and 2.7 ± 0.74-fold (*P* ≤ 0.05) elevation of phosphorylated AMPK and ACC, respectively, at a concentration of 0.4 mmol/l. In contrast, Akt phosphorylation was unaffected by rHDL (1.12 ± 0.27-fold), while insulin (100 nmol/l) had a 51 ± 12.5-fold (*P* ≤ 0.01) effect (Fig. 3E, F). Representative immunoblots of those quantified in Fig. 3A, C, and E are given in Fig. 3B, D, and F, respectively.

### C-terminal 190–243 peptide of lipid-free apoA-I increases glucose uptake in skeletal muscle

**Fig. 4.**
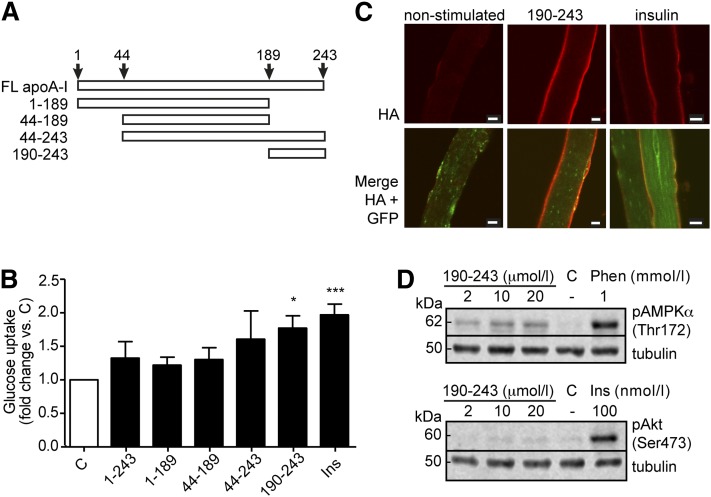
Effect of specific regions of apoA-I on glucose uptake. (A) Schematic linear presentation of apoA-I fragments analyzed for their potency in inducing glucose uptake. FL, full length. (B) Glucose uptake was measured in L6 myotubes, which had been serum starved for 2 h then stimulated with the peptides representing residues 1–243, 1–189, 44–189, 44–243, or 190–243 of apoA-I at equimolar particle concentrations (1 µmol/l), or 100 nmol/l insulin (Ins), or nonstimulated (C) for 60 min. Peptide treatments were based on mono- or dimeric organization for 1–243, 1–189, 44–189, and 44–243 (1 µmol/l peptide unit), and di- or tetrameric for 190–243 (2 µmol/l peptide unit). n = 3–4; *P ≤ 0.05, ***P ≤ 0.001. (C) Immunofluorescence images of FDB fibers from the HA-GLUT4-GFP transgenic mouse treated ex vivo with 190–243 fragment of apoA-I (2 µmol/l) insulin (100 nmol/l) or nonstimulated for 60 min. Size bars are 10 µm. Images are indicative of three independent experiments with FDB fibers from 2–4 mice per experiment. (D) Myotubes were serum starved for 4 h then treated for 60 min with 2, 10, or 20 µmol/l of 190–243 peptide, 1 mmol/l phenformin, 100 nmol/l insulin, or control (C) followed by western blot analysis for phosphorylated AMPK (upper panel) or phosphorylated Akt (lower panel). Tubulin was used as loading control. Blots shown are representative of three independent experiments.

The relative effect of specific regions of apoA-I to increase glucose uptake was investigated using full-length and truncated protein fragments corresponding to residues 1–243 (full-length), 1–189 (N-terminal/central domain), 44–189 (central domain), 44–243 (central/C-terminal domain), and 190–243 (C-terminal domain) of full-length apoA-I (**Fig. 4A**). As can be seen in Fig. 4B, all five peptides induced glucose uptake, with peptide fragment 190–243 displaying the largest and most consistent influence on L6 myotube glucose uptake (1.77 ± 0.23-fold change versus control; *P* ≤ 0.05). To verify this observation, confocal immunofluorescence imaging of FDB fibers labeled with HA-antibody was performed after ex vivo incubation with the 190–243 peptide. Relative to basal conditions, these images show greater membrane levels of GLUT4 protein in response to the 190–243 peptide (Fig. 4C), thus supporting the findings in Fig. 4B. We next used western blotting to examine the signaling pathway activated by the 190–243 peptide. Incubation of L6 myotubes with increasing concentrations (2, 10, and 20 µmol/l) of 190–243 peptide resulted in phosphorylation of AMPK (Fig. 4D). In contrast, no Akt phosphorylation was observed. Phenformin at 1 mmol/l and insulin at 100 nmol/l were used as positive controls for phosphorylation of AMPK and Akt, respectively. Finally, our initial analyses on lipid-free apoA-I-induced signaling in L6 myotubes treated with liver X receptor (LXR) agonist to induce overexpression of ABCA1 suggest a non-ABCA1-dependent signaling pathway (data not shown).

### Structural conformation of lipid-free 190–243 fragment resembles the conformation of lipid-bound apoA-I

**Fig. 5.**
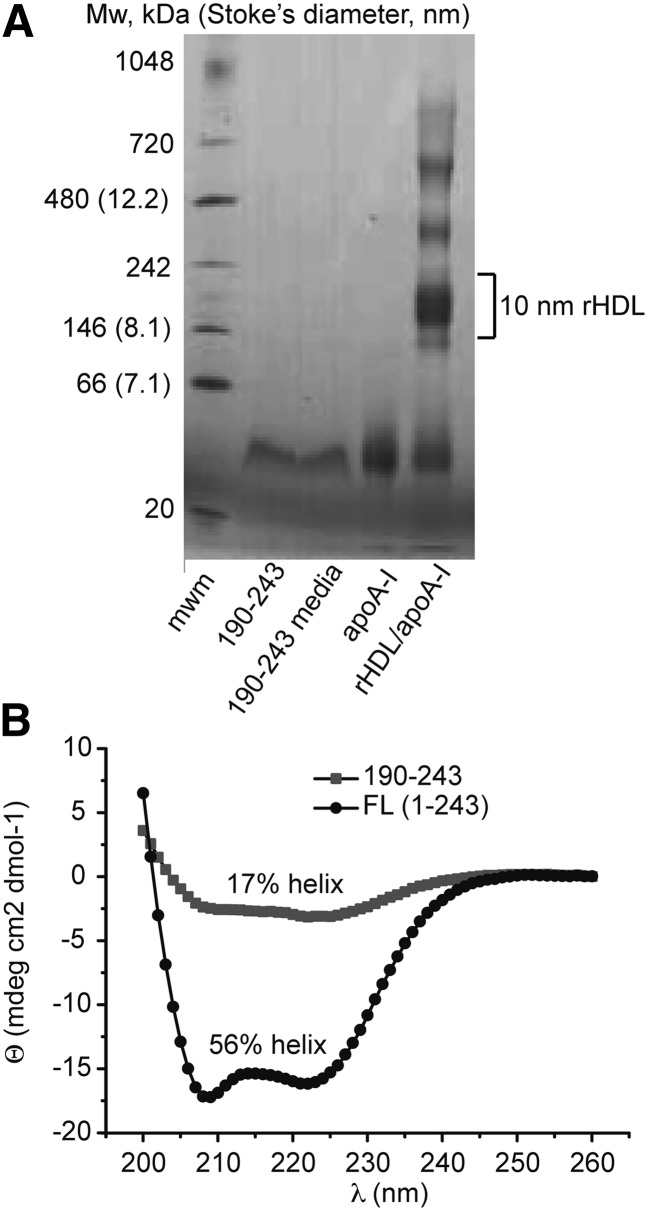
Properties of 190–243 apoA-I fragment. (A) Native gel analysis of 190–243 fragment in solution (190–243, 0.26 µg) and after incubation with L6 myotubes for 60 min (190–243 media, 0.26 µg). Lipid-free apoA-I (apo A-I, 4.4 µg) and rHDL (apoA-I, 9.4 µg) are used as reference samples. The results shown are representative of five independent experiments. (B) CD spectra of full-length (FL) apoA-I (black circles) and 190–243 fragment (gray squares) analyzed in PBS buffer solution at a protein concentration of 0.1 mg/ml.

We hypothesized that binding of cellular lipids to the 190–243 fragment upon incubation with L6 myotubes and FDB fibers may be necessary for its glucose uptake-inducing effect. It is known that the 190–243 fragment can promote cholesterol efflux from cultured cells and form discoidal particles by solubilization of lipid in solution (21, 22), which can be visualized as oligomers on native PAGE. To assess oligomer formation indicative of lipid binding, purified 190–243 peptide and conditioned media from cells treated with the 190–243 fragment for 1 h were run on a blue native PAGE and Coomassie stained (**Fig. 5A**). Under both conditions, 190–243 appeared as a single band at approximately 40 kDa corresponding to a tetramer. Lipid-free apoA-I and rHDL were included on the gel as a full-length protein monomer and dimer reference. Although the presence of minute amounts of lipids in the 190–243 tetramers cannot be excluded, the migration distance is clearly different from the rHDL particles formed by the 190–243 peptide in interaction with cultured baby hamster kidney (BHK) cells expressing human ABCA1 (∼10 nm rHDL formed; approximately corresponding to the 242 kDa marker protein) and from interaction with phospholipid multilamellar vesicles (∼17 nm rHDL) (21). Moreover, our findings on the oligomeric state of the 190–243 peptide is in agreement with those on the 198–243 peptide that self-associates as tetramers in solution (23).

As depicted in Fig. 1, the structure of apoA-I in the apo-state is significantly different from the structural organization of the same protein in rHDL particles. This structural transition of the lipid-binding process involves a major increase in α-helical secondary structure (from about 44–55% in the apo-state to 78% α-helical secondary structure in discoidal HDL) (17, 24). As rHDL is potent in stimulation of glucose uptake in myotubes, we speculated that lipid-free 190–243 fragment may adopt an amphiphatic α-helix in solution. To investigate this, CD spectroscopy spectra were obtained at a protein concentration of 0.1 mg/ml for the 190–243 fragment and full-length protein for comparison (Fig. 5). The helical content was estimated from their molar ellipticities at 222 nm to be 17% (or 21% at 0.2 mg/ml; data not shown) and 56% for 190–243 fragment and full-length apoA-I, respectively, suggesting an increase in helical structure of the fragment (see Discussion).

## DISCUSSION

This study analyzed the capability of discoidal HDL and the potency of subdomains of apoA-I to promote translocation of the GLUT4 glucose transporter to the plasma membrane and thereby induce glucose uptake. From our work, it is clear that discoidal HDL promotes glucose uptake in cultured skeletal muscle, eliciting an effect comparable to insulin. Furthermore, we have made the discovery that the 190–243 peptide, corresponding to the C-terminal domain of apoA-I, is itself an efficient agonist for glucose uptake.

Currently only two studies have shown that both lipid-free apoA-I and mature spherical HDL can increase glucose uptake in skeletal muscle via the AMPK signaling pathway (6, 7). However, what had not been clearly addressed was the efficacy of discoidal HDL, an important consideration given the marked alterations in structure that apoA-I undergoes during HDL maturation (depicted in Fig. 1). While the study by Drew et al. (7) found increased ACC phosphorylation in muscle biopsies after 4 h rHDL infusion, indicative of AMPK activation, it is difficult to specifically attribute this observation to discoidal HDL. As the authors acknowledge, rHDL is likely to become rapidly remodeled to mature HDL in the plasma. By using both synthetic discoidal HDL containing different phospholipid species and phospholipid vesicles lacking an apoprotein component, we were able to show that the glucose uptake response to rHDL is apoA-I dependent. Whether this involves lipid exchange of rHDL with the L6 myotubes, a capability that the phospholipid vesicles essentially lack, is not clear. Although rHDL does not reflect the physiological heterogeneity of nascent discoidal HDL, rHDL offers the advantage of allowing defined oligomeric states to be studied and is free from plasma contaminants and modifications, such as oxidation and glycation, which have been shown to influence HDL function (25, 26). Despite the use of a supraphysiological concentration of apoA-I, our signaling data in rHDL-treated L6 myotubes also concur with previous findings that showed activation of AMPK and its downstream target ACC but found no increase in Akt phosphorylation. This is an important feature of apoA-I/HDL action in muscle that allows glucose uptake to be maintained independently of the insulin signaling pathway.

Evidence for the direct effect of apoA-I in its lipid-free and various lipid-bound states on skeletal muscle glucose uptake is now strong, having been verified in murine C2C12 myotubes (6), primary human cultures from diabetic patients (7), and now in rat L6 myotubes. Using myofibers from the HA-GLUT4-GFP transgenic mouse, this study has added a further, highly sensitive model that confirms the direct influence of discoidal HDL on GLUT4 plasma membrane levels.

To exploit the properties of apoA-I for therapeutic means, we initiated the search for unique functional peptides by analyzing the glucose uptake effect of defined regions of the apoA-I protein. It was evident that peptides containing the C-terminal domain had an improved capacity to induce glucose uptake in relation to peptides lacking this region and that the 190–243 fragment alone carried the most potent effect. It is interesting to note that structural studies reveal that this region carries the highest lipid-binding capacity in relation to N-terminal and central domains (23, 27) and that a 198–243 peptide self-associates into a tetrameric organization (23). Indeed, lipid binding is impaired in an apoA-I mutant lacking this region (21, 28), whereas this fragment alone can initiate cholesterol efflux from J774 macrophages that have been stimulated to produce high ABCA1 levels and can also lead to formation of peptide/lipid particles with apparent diameters of 10 or 17 nm depending on whether they have been incubated with multilamellar vesicles or BHK cells (21). ApoA-I is known to interact with ABCA1, with cholesterol efflux resulting (14). While we cannot completely exclude the possibility that minor amounts of cholesterol and phospholipids are taken up by the tetrameric peptide, the native gel analysis clearly show that discoidal-like HDL particles are not formed. In conclusion, we observe no evidence of lipid binding to the 190–243 peptide after incubation with L6 myotubes, allowing us to speculate that the glucose uptake effect of this peptide is independent of lipid efflux.

This picture is in partial agreement with Drew et al (7) who, despite showing lipid efflux to HDL, found this to be independent of ABCA1-dependent induced glucose uptake. This is perfectly feasible on the basis of Vedhachalam et al. (28) who proposed a two-step process of lipid efflux to apoA-I, which involves ABCA1 binding followed by membrane lipid site association. It is possible that in skeletal muscle, the 190–243 fragment binds ABCA1 but does not continue to the second step of binding to the membrane domain, perhaps due to internalization, an event known to be necessary for the AMPK signaling and glucose uptake effects of lipid-free apoA-I (6).

It is notable that in our experiments apoA-I shows a clear trend toward increasing glucose uptake, but in contrast to other studies (6, 7), it is not significant, whereas rHDL and the 190–243 peptide produce significant insulin-like effects. We speculated that the 190–243 fragment better resembles the effect of full-length apoA-I in rHDL than in the apo-state due to structural rearrangements of the peptide. To analyze this, we used CD spectroscopy to compare secondary structure contents of intact apoA-I protein and the peptide fragment.

**Fig. 6.**
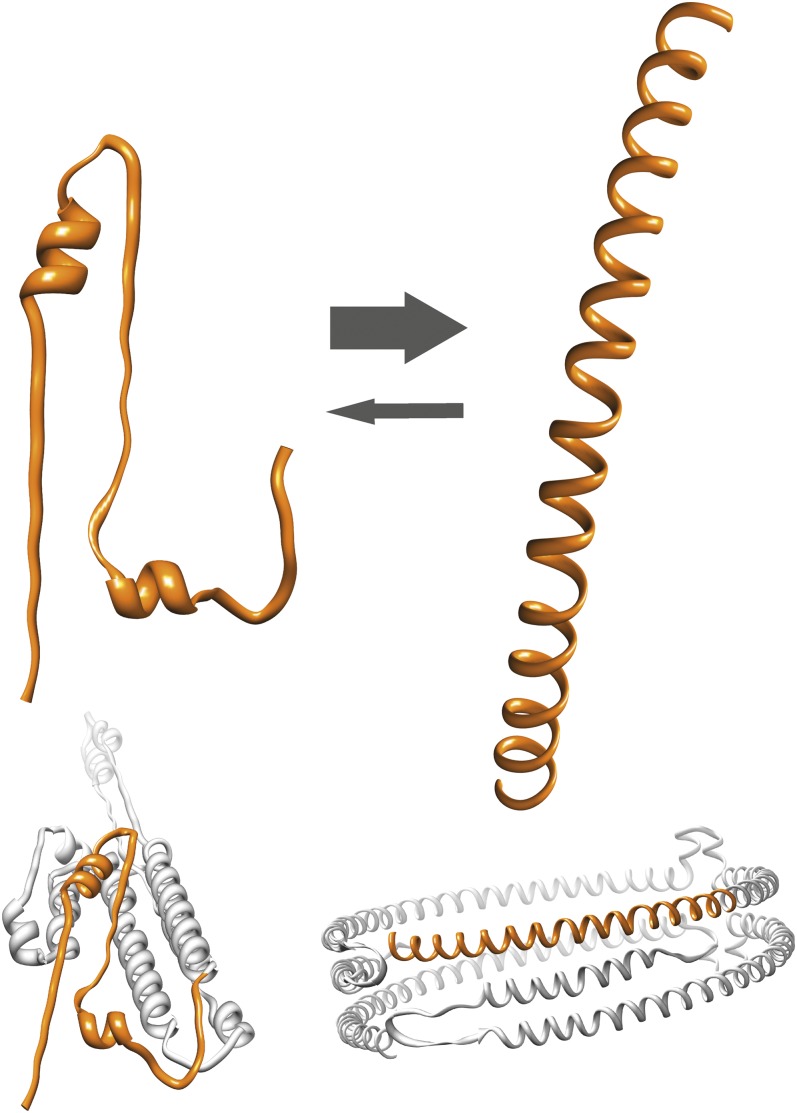
Lipid-free apoA-I 190–243 fragment may adopt a structure similar to that in rHDL. (Left) Structure of 190–243 fragment (top) as folded in the lipid-free structure model (bottom) (36). (Right) Structure of the 190–243 fragment as an extended amphipathic α-helix (top) corresponding to the structural arrangement of this region of apoA-I in discoidal HDL (bottom) (16). Arrows indicate a shift toward HDL-resembling amphipathic α-helical structure of lipid-free 190–243 apoA-I fragments at equilibrium in solution.

In the apo-state, this region of the intact protein contains no helical structure (29), and the obtained 17–21% average α-helical content of the 190–243 fragment thus means a net increase in helical structure of this region. Under certain conditions, this may be even higher as reported in previous analyses (α-helical content is 33 ± 3% in Ref. 22). The results suggest that the 190–243 fragment in the apo-state adopts an rHDL-resembling structure in solution (**Fig. 6**) that, similar to the previously described 198–243 peptide, adopts a tetrameric organization (23); we therefore propose that this structural organization is the preferred structure to induce glucose uptake in skeletal muscle.

Although a role for the ABCA1 receptor has been shown for HDL and lipid-free apoA-I-induced glucose uptake (7), this does not exclude the involvement of alternative receptors. A potential candidate is the ecto-F1-ATPase, which has been shown to act as an apoA-I receptor in hepatocytes (30), adipocytes (31), and endothelial cells (32, 33), but this interaction is yet to be characterized in skeletal muscle. The possibility of novel yet unidentified receptors for apoA-I also remains. Our discovery of the novel action of the 190–243 peptide provides a valuable tool for future investigations aimed at identifying novel receptor targets. Such work will not only help to refine mechanisms of apoA-I/HDL action, but will themselves represent exciting pharmacological targets.

In conclusion, this study has filled the gap in knowledge regarding the effect of discoidal HDL on skeletal muscle glucose uptake, showing that the activity of apoA-I in this protein arrangement is as potent as it is in the lipid-free and mature spherical HDL states. We have also identified the 190–243 peptide as harboring the relevant characteristics required to elicit this effect. From the example of apoA-I mimetics currently under development in the field of cardiovascular disease (34), the production and testing of smaller therapeutic peptides derived from knowledge of the 190–243 fragment may provide promise, with the apoA-I mimetic L-4F preventing insulin resistance in vivo via effects on insulin signaling and lipid metabolism (35). Future work is now needed on small peptide regions of the 190–243 fragment, which may prove to be viable treatment agents for insulin resistance and type 2 diabetes.

## References

[1] Zannis V. I. Chroni A. Krieger M. 2006 Role of apoA-I, ABCA1, LCAT, and SR-BI in the biogenesis of HDL. J. Mol. Med. (Berl.). 84: 276–294 16501936 10.1007/s00109-005-0030-4

[2] Rye K. A. Bursill C. A. Lambert G. Tabet F. Barter P. J. 2009 The metabolism and anti-atherogenic properties of HDL. J. Lipid Res. 50(Suppl.): S195–S200 19033213 10.1194/jlr.R800034-JLR200 PMC2674714

[3] Despres J. P. Lemieux I. Dagenais G. R. Cantin B. Lamarche B. 2000 HDL-cholesterol as a marker of coronary heart disease risk: the Quebec cardiovascular study. Atherosclerosis. 153: 263–272 11164415 10.1016/s0021-9150(00)00603-1

[4] Gatti A. Maranghi M. Bacci S. Carallo C. Gnasso A. Mandosi E. Fallarino M. Morano S. Trischitta V. Filetti S. 2009 Poor glycemic control is an independent risk factor for low HDL cholesterol in patients with type 2 diabetes. Diabetes Care. 32: 1550–1552 19487641 10.2337/dc09-0256 PMC2713640

[5] Laakso M. 1999 Hyperglycemia and cardiovascular disease in type 2 diabetes. Diabetes. 48: 937–942 10331395 10.2337/diabetes.48.5.937

[6] Han R. Lai R. Ding Q. Wang Z. Luo X. Zhang Y. Cui G. He J. Liu W. Chen Y. 2007 Apolipoprotein A-I stimulates AMP-activated protein kinase and improves glucose metabolism. Diabetologia. 50: 1960–1968 17639303 10.1007/s00125-007-0752-7

[7] Drew B. G. Duffy S. J. Formosa M. F. Natoli A. K. Henstridge D. C. Penfold S. A. Thomas W. G. Mukhamedova N. de Courten B. Forbes J. M. 2009 High-density lipoprotein modulates glucose metabolism in patients with type 2 diabetes mellitus. Circulation. 119: 2103–2111 19349317 10.1161/CIRCULATIONAHA.108.843219

[8] Zhang Q. Zhang Y. Feng H. Guo R. Jin L. Wan R. Wang L. Chen C. Li S. 2011 High density lipoprotein (HDL) promotes glucose uptake in adipocytes and glycogen synthesis in muscle cells. PLoS ONE. 6: e23556 21886796 10.1371/journal.pone.0023556 PMC3158770

[9] Drew B. G. Rye K. A. Duffy S. J. Barter P. Kingwell B. A. 2012 The emerging role of HDL in glucose metabolism. Nat. Rev. Endocrinol. 8: 237–245 22271188 10.1038/nrendo.2011.235

[10] Tremblay F. Dubois M. J. Marette A. 2003 Regulation of GLUT4 traffic and function by insulin and contraction in skeletal muscle. Front. Biosci. 8: d1072–d1084 12957810 10.2741/1137

[11] Towler M. C. Hardie D. G. 2007 AMP-activated protein kinase in metabolic control and insulin signaling. Circ. Res. 100: 328–341 17307971 10.1161/01.RES.0000256090.42690.05

[12] Mu J. Brozinick J. T. Jr Valladares O. Bucan M. Birnbaum M. J. 2001 A role for AMP-activated protein kinase in contraction- and hypoxia-regulated glucose transport in skeletal muscle. Mol. Cell. 7: 1085–1094 11389854 10.1016/s1097-2765(01)00251-9

[13] Musi N. Fujii N. Hirshman M. F. Ekberg I. Froberg S. Ljungqvist O. Thorell A. Goodyear L. J. 2001 AMP-activated protein kinase (AMPK) is activated in muscle of subjects with type 2 diabetes during exercise. Diabetes. 50: 921–927 11334434 10.2337/diabetes.50.5.921

[14] Favari E. Calabresi L. Adorni M. P. Jessup W. Simonelli S. Franceschini G. Bernini F. 2009 Small discoidal pre-beta1 HDL particles are efficient acceptors of cell cholesterol via ABCA1 and ABCG1. Biochemistry. 48: 11067–11074 19839639 10.1021/bi901564g

[15] Lagerstedt J. O. Budamagunta M. S. Oda M. N. Voss J. C. 2007 Electron paramagnetic resonance spectroscopy of site-directed spin labels reveals the structural heterogeneity in the N-terminal domain of apoA-I in solution. J. Biol. Chem. 282: 9143–9149 17204472 10.1074/jbc.M608717200

[16] Lagerstedt J. O. Cavigiolio G. Budamagunta M. S. Pagani I. Voss J. C. Oda M. N. 2011 Structure of apolipoprotein A-I N terminus on nascent high density lipoproteins. J. Biol. Chem. 286: 2966–2975 21047795 10.1074/jbc.M110.163097 PMC3024791

[17] Petrlova J. Duong T. Cochran M. C. Axelsson A. Morgelin M. Roberts L. M. Lagerstedt J. O. 2012 The fibrillogenic L178H variant of apolipoprotein A-I forms helical fibrils. J. Lipid Res. 53: 390–398 22184756 10.1194/jlr.M020883 PMC3276462

[18] Morrow J. A. Segall M. L. Lund-Katz S. Phillips M. C. Knapp M. Rupp B. Weisgraber K. H. 2000 Differences in stability among the human apolipoprotein E isoforms determined by the amino-terminal domain. Biochemistry. 39: 11657–11666 10995233 10.1021/bi000099m

[19] Fazakerley D. J. Lawrence S. P. Lizunov V. A. Cushman S. W. Holman G. D. 2009 A common trafficking route for GLUT4 in cardiomyocytes in response to insulin, contraction and energy-status signalling. J. Cell Sci. 122: 727–734 19208760 10.1242/jcs.041178 PMC2720923

[20] Lizunov V. A. Stenkula K. G. Lisinski I. Gavrilova O. Yver D. R. Chadt A. Al-Hasani H. Zimmerberg J. Cushman S. W. 2012 Insulin stimulates fusion, but not tethering, of GLUT4 vesicles in skeletal muscle of HA-GLUT4-GFP transgenic mice. Am. J. Physiol. Endocrinol. Metab. 302: E950–E960 22297303 10.1152/ajpendo.00466.2011 PMC3330721

[21] Vedhachalam C. Chetty P. S. Nickel M. Dhanasekaran P. Lund-Katz S. Rothblat G. H. Phillips M. C. 2010 Influence of apolipoprotein (Apo) A-I structure on nascent high density lipoprotein (HDL) particle size distribution. J. Biol. Chem. 285: 31965–31973 20679346 10.1074/jbc.M110.126292 PMC2952197

[22] Tanaka M. Koyama M. Dhanasekaran P. Nguyen D. Nickel M. Lund-Katz S. Saito H. Phillips M. C. 2008 Influence of tertiary structure domain properties on the functionality of apolipoprotein A-I. Biochemistry. 47: 2172–2180 18205410 10.1021/bi702332b

[23] Zhu H. L. Atkinson D. 2007 Conformation and lipid binding of a C-terminal (198–243) peptide of human apolipoprotein A-I. Biochemistry. 46: 1624–1634 17279626 10.1021/bi061721z PMC2518689

[24] Saito H. Dhanasekaran P. Nguyen D. Deridder E. Holvoet P. Lund-Katz S. Phillips M. C. 2004 Alpha-helix formation is required for high affinity binding of human apolipoprotein A-I to lipids. J. Biol. Chem. 279: 20974–20981 15020600 10.1074/jbc.M402043200

[25] Bergt C. Pennathur S. Fu X. Byun J. O'Brien K. McDonald T. O. Singh P. Anantharamaiah G. M. Chait A. Brunzell J. 2004 The myeloperoxidase product hypochlorous acid oxidizes HDL in the human artery wall and impairs ABCA1-dependent cholesterol transport. Proc. Natl. Acad. Sci. USA. 101: 13032–13037 15326314 10.1073/pnas.0405292101 PMC516512

[26] Nobecourt E. Tabet F. Lambert G. Puranik R. Bao S. Yan L. Davies M. J. Brown B. E. Jenkins A. J. Dusting G. J. 2010 Nonenzymatic glycation impairs the antiinflammatory properties of apolipoprotein A-I. Arterioscler. Thromb. Vasc. Biol. 30: 766–772 20110571 10.1161/ATVBAHA.109.201715 PMC3038672

[27] Palgunachari M. N. Mishra V. K. Lund-Katz S. Phillips M. C. Adeyeye S. O. Alluri S. Anantharamaiah G. M. Segrest J. P. 1996 Only the two end helixes of eight tandem amphipathic helical domains of human apo A-I have significant lipid affinity. Implications for HDL assembly. Arterioscler. Thromb. Vasc. Biol. 16: 328–338 8620350 10.1161/01.atv.16.2.328

[28] Vedhachalam C. Duong P. T. Nickel M. Nguyen D. Dhanasekaran P. Saito H. Rothblat G. H. Lund-Katz S. Phillips M. C. 2007 Mechanism of ATP-binding cassette transporter A1-mediated cellular lipid efflux to apolipoprotein A-I and formation of high density lipoprotein particles. J. Biol. Chem. 282: 25123–25130 17604270 10.1074/jbc.M704590200

[29] Chetty P. S. Mayne L. Lund-Katz S. Stranz D. Englander S. W. Phillips M. C. 2009 Helical structure and stability in human apolipoprotein A-I by hydrogen exchange and mass spectrometry. Proc. Natl. Acad. Sci. USA. 106: 19005–19010 19850866 10.1073/pnas.0909708106 PMC2776417

[30] Martinez L. O. Jacquet S. Esteve J. P. Rolland C. Cabezon E. Champagne E. Pineau T. Georgeaud V. Walker J. E. Terce F. 2003 Ectopic beta-chain of ATP synthase is an apolipoprotein A-I receptor in hepatic HDL endocytosis. Nature. 421: 75–79 12511957 10.1038/nature01250

[31] Howard A. D. Verghese P. B. Arrese E. L. Soulages J. L. 2011 The beta-subunit of ATP synthase is involved in cellular uptake and resecretion of apoA-I but does not control apoA-I-induced lipid efflux in adipocytes. Mol. Cell. Biochem. 348: 155–164 21069432 10.1007/s11010-010-0650-z PMC3071259

[32] Radojkovic C. Genoux A. Pons V. Combes G. de Jonge H. Champagne E. Rolland C. Perret B. Collet X. Terce F. 2009 Stimulation of cell surface F1-ATPase activity by apolipoprotein A-I inhibits endothelial cell apoptosis and promotes proliferation. Arterioscler. Thromb. Vasc. Biol. 29: 1125–1130 19372457 10.1161/ATVBAHA.109.187997

[33] Cavelier C. Ohnsorg P. M. Rohrer L. von Eckardstein A. 2012 The beta-chain of cell surface F(0)F(1) ATPase modulates apoA-I and HDL transcytosis through aortic endothelial cells. Arterioscler. Thromb. Vasc. Biol. 32: 131–139 21979433 10.1161/ATVBAHA.111.238063

[34] Getz G. S. Reardon C. A. 2011 Apolipoprotein A-I and A-I mimetic peptides: a role in atherosclerosis. *J. Inflammation Res.* **2011:** 83–92

[35] Peterson S. J. Kim D. H. Li M. Positano V. Vanella L. Rodella L. F. Piccolomini F. Puri N. Gastaldelli A. Kusmic C. 2009 The L-4F mimetic peptide prevents insulin resistance through increased levels of HO-1, pAMPK, and pAKT in obese mice. J. Lipid Res. 50: 1293–1304 19224872 10.1194/jlr.M800610-JLR200 PMC2694329

[36] Lagerstedt J. O. Budamagunta M. S. Liu G. S. Devalle N. C. Voss J. C. Oda M. N. 2012 The “beta-clasp” model of apolipoprotein A-I--a lipid-free solution structure determined by electron paramagnetic resonance spectroscopy. Biochim. Biophys. Acta. 1821: 448–455 22245143 10.1016/j.bbalip.2011.12.010 PMC3402359

[37] Silva R. A. Huang R. Morris J. Fang J. Gracheva E. O. Ren G. Kontush A. Jerome W. G. Rye K. A. Davidson W. S. 2008 Structure of apolipoprotein A-I in spherical high density lipoproteins of different sizes. Proc. Natl. Acad. Sci. USA. 105: 12176–12181 18719128 10.1073/pnas.0803626105 PMC2527885

